# Triple Expressor Lymphoma: Presentation With Acute Paraparesis Due to Spinal Cord Compression

**DOI:** 10.7759/cureus.31237

**Published:** 2022-11-08

**Authors:** Joseph Alburqueque-Melgarejo, Juan Carlos Roque-Quezada, Horus Michael Virú-Flores, Emanuel Salcedo Davila, Javier Dulanto Moscoso, Jhony Alberto De la Cruz-Vargas

**Affiliations:** 1 Biomedical Sciences Research Institute, Faculty of Human Medicine of the Ricardo Palma University, Ricardo Palma University, Lima, PER; 2 Faculty of Human Medicine, San Juan Bautista Private University, Lima, PER; 3 Department of Medical Education, Ricardo Palma University, Lima, PER; 4 Department of Medical Hematology, Navy Medical Center, Lima, PER

**Keywords:** triple hit lymphoma, triple expressor lymphoma, spinal cord compression, non-hodgkin’s lymphoma, diffuse large b-cell lymphoma

## Abstract

Diffuse large B-cell lymphoma (DBLCL) is the most common type of non-Hodgkin's lymphoma (NHL). Triple expressor lymphoma is a subgroup of non-Hodgkin's lymphomas that exhibits simultaneous overexpression of the MYC, BCL2, and BCL6 genes. This entity is characterized by its aggressive behavior and its poor response to chemotherapy regimens traditionally used, such as the standard R-CHOP (rituximab plus cyclophosphamide, doxorubicin, vincristine, and prednisone) regimen. This neoplasm can have varied clinical manifestations according to its initial location and usually has central nervous system (CNS) involvement. This article presents the case of a triple expressor lymphoma with spinal involvement at the level of the thoracic vertebrae in a previously healthy 34-year-old female patient, which lead to acute paraparesis due to spinal cord compression. Nevertheless, appropriate treatment with the DA-EPOCH-R (dose-adjusted etoposide, prednisone, vincristine, cyclophosphamide, doxorubicin, and rituximab) regimen resulted in the recovery of the motor and sensory function of the lower extremities.

## Introduction

Diffuse large B-cell lymphoma (DLBCL) accounts for approximately 30% to 40% of non-Hodgkin's lymphoma (NHL) cases being the most frequent type with an estimated incidence of 77,240 new cases in 2021. This neoplasm comprises a heterogeneous group of biologically distinct entities resulting from the clonal proliferation of a germinal center or post-germinal center B lymphocytes and that present different biological, clinical, and treatment response features. These can be classified according to their cell of origin or by their molecular characteristics [1,2].

Some DLBCL present genetic mutations that lead to the overexpression of oncogenic proteins. The most frequently involved genes are MYC, BCL-2, and BCL-6. Recently, the World Health Organization (WHO) in the 2016 classification of lymphoid neoplasms has proposed the category of high-grade B-cell lymphomas with rearrangements in the MYC, BCL-2, and/or BCL-6 genes, which groups to double-expressor/double-hit and triple-expressor/triple-hit lymphomas, according to whether they present overexpression of the aforementioned genes or if they present chromosomal rearrangements. Triple expressor lymphoma is the DLBCL that expresses the three genes previously mentioned, but it does not present chromosomal rearrangements, and it usually has a poor prognosis, which is why different therapeutic options are required [2,3]. This article presents the case of a 34-year-old female patient who presented to the hospital emergency room with acute low back pain and decreased strength in the lower extremities, whose CT scan revealed a mass infiltrating the thoracic vertebrae and causing spinal cord compression. The biopsy revealed a triple expressor lymphoma.

## Case presentation

A 34-year-old female patient presented to the hospital emergency room with sudden-onset low back pain and bilateral motor and sensory deficits in the lower extremities. Prior medical history was not contributory. However, the patient had a history of lumbar pain of approximately four months of evolution, dull in nature, and not reproducible with touch, which initially subsided with the administration of analgesics, and subsequently became more severe and refractory to analgesics. On questioning, the patient admitted that, in addition to the pain, she had noticed a decrease in appetite and unwanted weight loss. The patient denied fever or night sweats.

Physical examination revealed decreased muscle strength (1/5) in the lower limbs, areflexia, and decreased sensitivity. There was no evidence of loss of control of the bladder and anal sphincters. Laboratory tests on admission showed anemia (hemoglobin: 12.3 g/dL), mild leukocytosis (11.18 x103 cells/mm3), and elevated levels of lactate dehydrogenase (LDH: 2270 U/L). Serology for HIV, human T-lymphotropic viruses (HTLV)-1/2, Epstein-Barr virus (EBV), and hepatitis B was negative. Laboratory findings on admission are summarized in Table 1.

**Table 1:** Laboratory investigations of the patient on admission

	Value	Reference range and units
Hemoglobin	10.4	12.3 – 16.3 g/dL
Hematocrit	31.8	39 – 52 %
Mean corpuscular volume (MCV)	77.3	80 – 100 fL
Mean corpuscular hemoglobin (MCH)	25.4	26 – 38 pg
Mean corpuscular hemoglobin concentration (MCHC)	32.8	31 – 37 g/dL
White blood cell count (WBC)	11.18	4 – 11 x10^3^ cells/mm^3^
Segmented neutrophils	8.99	2 – 7 x10^3^ cells/mm^3^
Bands	0.1	0 – 5 x10^3^ cells/mm^3^
Lactate dehydrogenase (LDH)	2270	135 - 225 U/L

**Figure 1:**
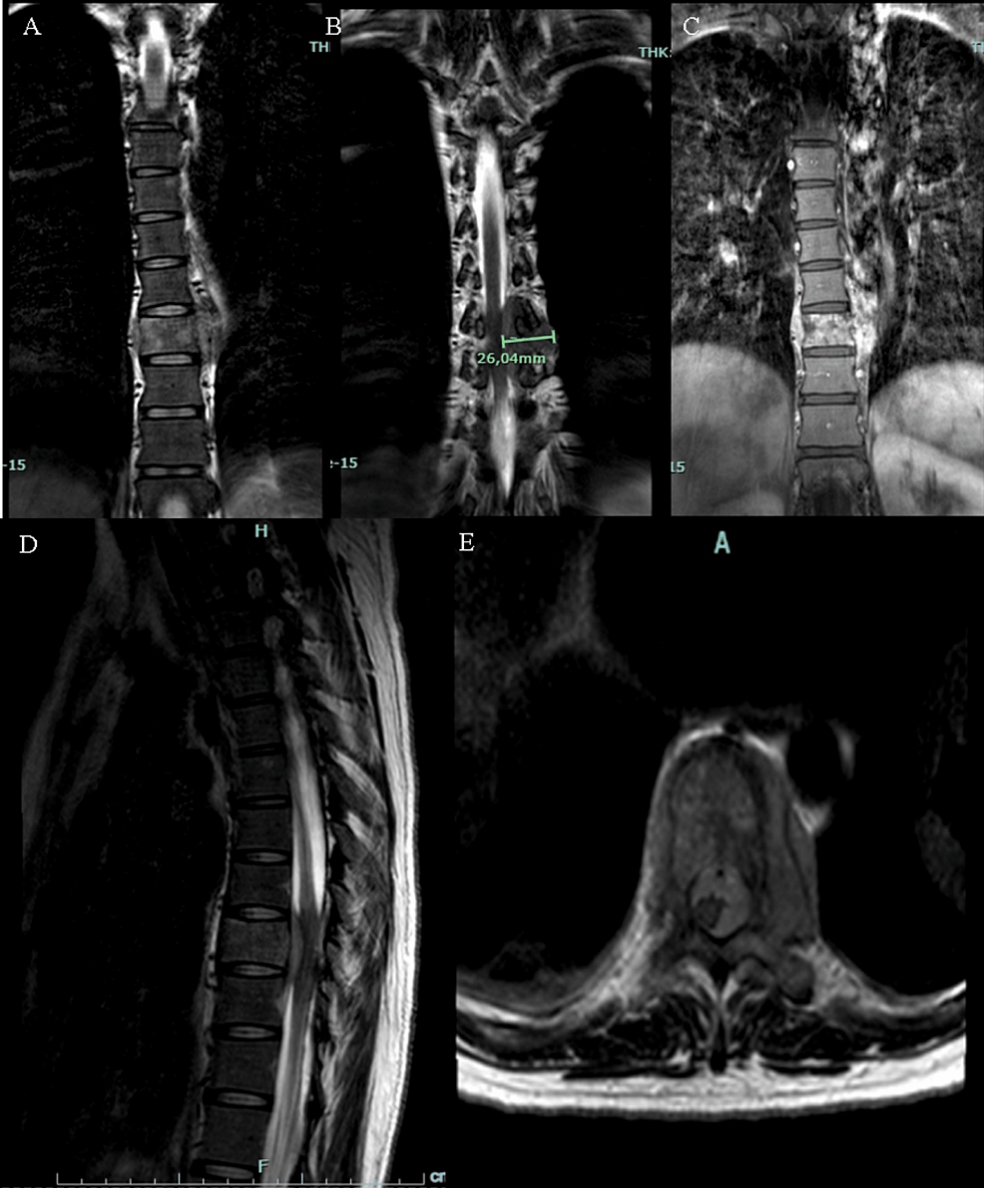
MRI of the thoracic and lumbar spine (A, B, C). A paravertebral lesion is observed at the level of the T9 thoracic vertebra with evidence of extension toward the epidural space and spinal cord compression predominantly on the left side. 1C shows a hyperintensity of the lesion in the T1W sequence. 1D shows a sagittal section showing a pathological fracture at the level of the T9 vertebral body with evidence of spinal cord compromise at that level. 1D shows an axial section at the level of the T9 vertebra. T1W: T1 weighted

During hospitalization, an MRI of the cervical, dorsal, and lumbar spine was performed, revealing a mass at the level of the T9 vertebra, which extended into the epidural space between the T8 and T10 spaces, with evidence of spinal cord compression. In addition, a paravertebral mass was present on the left side, approximately 36 x 26 mm in longitudinal and transverse diameter, with homogeneous enhancement after contrast administration and hyperintense on the T2 sequence (Figure 1).

The initial diagnosis was acute spinal cord compression syndrome at the T8 and T9 vertebrae levels due to an extradural neoplasm. Subsequently, the patient underwent an emergency laminectomy with decompression at the level of the T9 vertebra. The extracted surgical piece was sent to the pathological anatomy service for histopathological evaluation. In the postoperative period, the patient reported a notable improvement in muscle strength (from 1/5 to 3/5) and sensitivity of the lower extremities.

**Figure 2:**
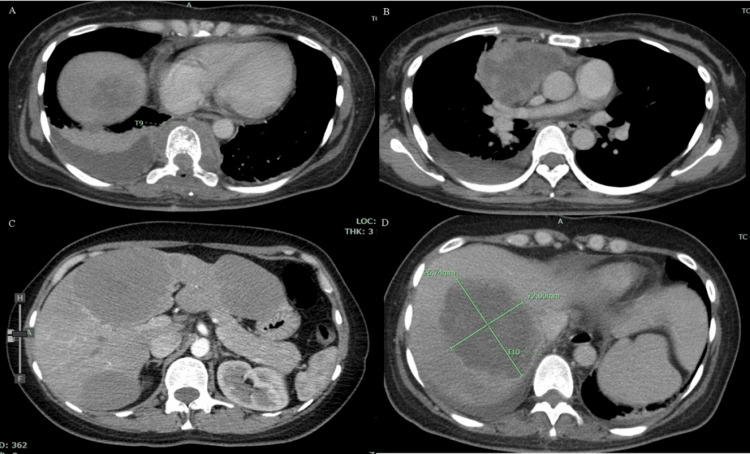
Contrast-enhanced computed tomography of the chest, abdomen, and pelvis 2A shows a mass at the level of the T9 vertebral body with paravertebral extension and the presence of pleural effusion on the right side. In addition, 2B shows a mediastinal mass with heterogeneous enhancement after contrast administration that displaces the heart and great vessels to the left side. 2C and 2D show multiple masses of variable sizes located in liver segments II, IV-A, V, VII, and VIII that show heterogeneous enhancement after contrast administration.

Contrast-enhanced computed tomography of the chest, abdomen, and pelvis revealed the presence of a mediastinal mass of approximately 10.5 cm x 6.6 cm x 6.9 cm in the longitudinal, anteroposterior, and transverse axis, respectively, with evidence of heterogeneous enhancement after contrast administration, displacing the heart and ascending aorta to the left. Adenopathies were also observed in the superior thoracic aperture of 9 x 6 mm and a right pleural effusion. In the abdomen, multiple masses were observed in the liver in segments II, IV-A, V and VII, and VIII, which showed heterogeneous enhancement with contrast administration. At the level of the T9 vertebra, an isodense paravertebral mass was observed with extension to the epidural space (Figure 2).

**Figure 3:**
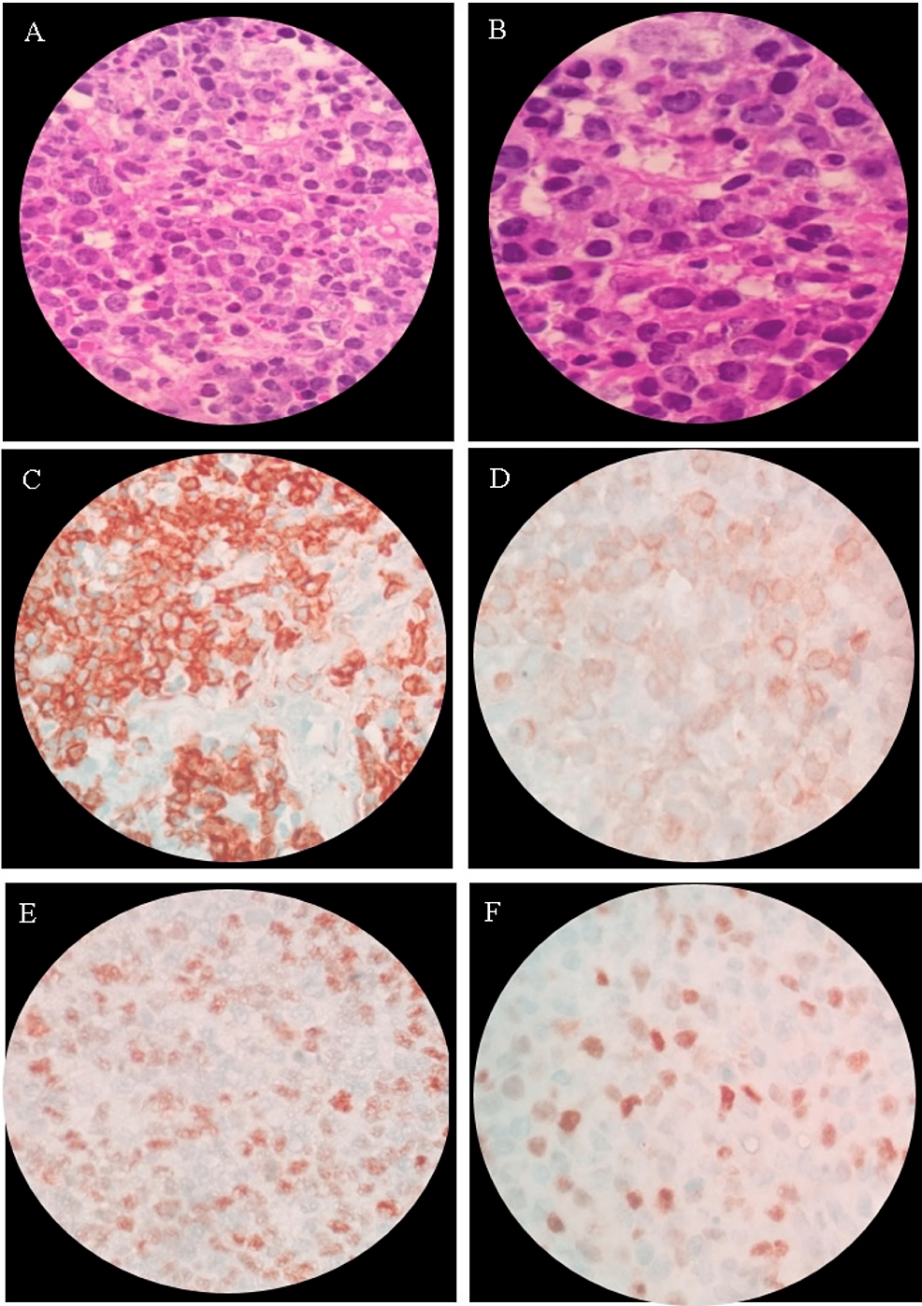
Histopathology of the lesion Histopathologic examination of the operative specimen showed a morphology consistent with germinal center (CG) diffuse large B-cell lymphoma (3A-3B). Immunohistochemistry revealed positivity for CD-20, BCL-2, BCL-6, and C-MYC (3C-3F).

**Figure 4:**
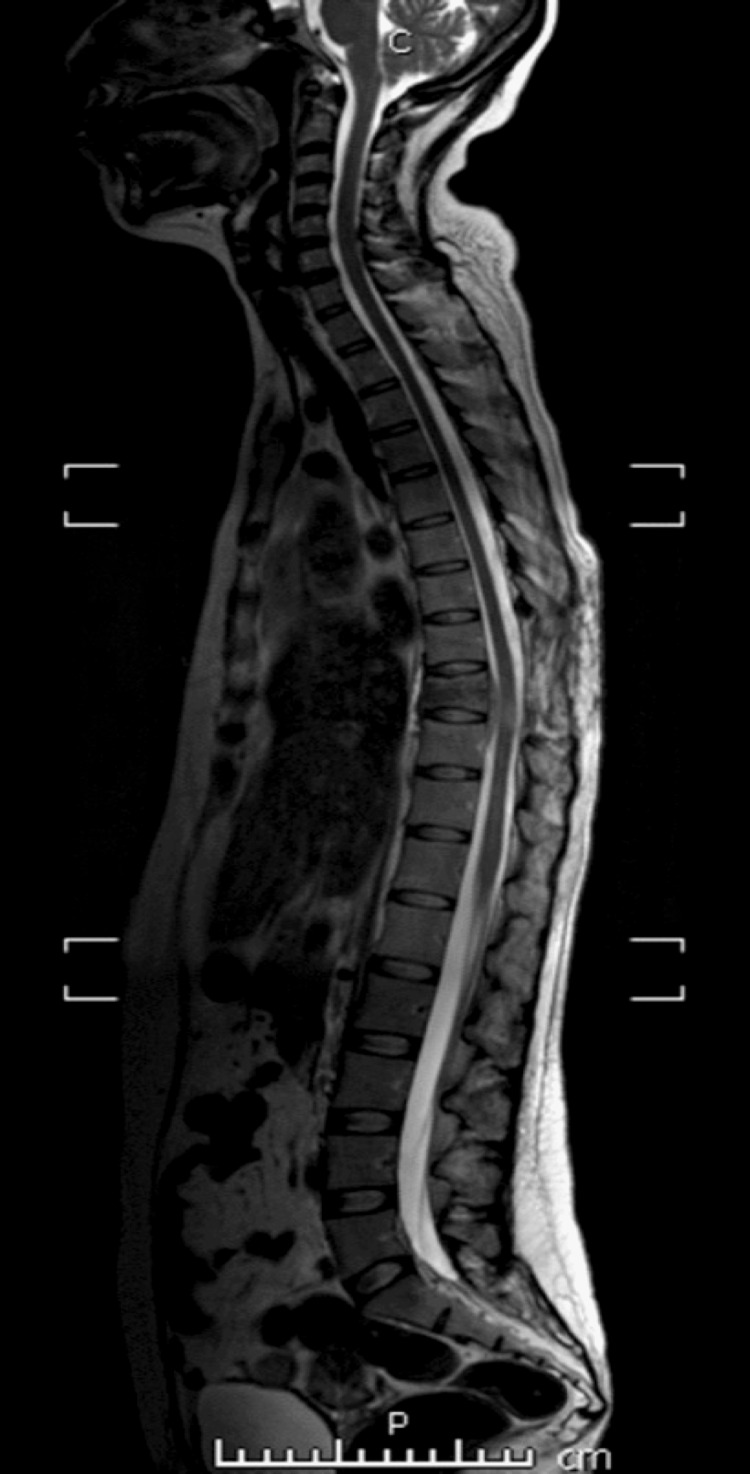
Magnetic resonance imaging of the spine after six cycles of chemotherapy with the DA-EPOCH-R regimen DA-EPOCH-R: dose-adjusted etoposide, prednisone, vincristine, cyclophosphamide, doxorubicin, and rituximab

Histopathologic examination of the surgical specimen showed a morphology consistent with germinal center B-cell subtype diffuse large B-cell lymphoma (GCB). Immunohistochemistry revealed positivity for CD20, BCL-2, BCL-6, and C-MYC. (Figure 3). However, no chromosomal rearrangements were observed on in situ hybridization. Furthermore, the index of proliferation Ki-67 was 80%. The patient was diagnosed with clinical stage IV triple expressor lymphoma, IPI: 3, and it was decided to start six cycles of chemotherapy with the DA-EPOCH-R regimen (etoposide 85 mg, adriamycin 17 mg, vincristine 0.7 mg, prednisone 204 mg, and rituximab 640 mg), after which the patient showed a marked improvement in neurological symptoms, as well as a decrease in the paravertebral mass (Figure 4). Prophylactic intrathecal chemotherapy with Ara-C 30 mg, Methotrexate 12 mg, and dexamethasone 4 mg was also administered. The patient is currently alive and in her fourth cycle of chemotherapy with the above-mentioned regimen. She presents a stationary evolution and continues to be followed up by an outpatient hematology clinic.

## Discussion

Diffuse large B-cell lymphoma (DLBCLs) is a neoplasm of medium to large B lymphocytes with a diffuse growth pattern and distortion of the lymph node architecture. This neoplasm comprises different entities with different biological, pathological, and clinical characteristics [4,5]. DLBCL has an estimated worldwide incidence of 150,000 new cases per year and accounts for approximately 30% to 40% of adult non-Hodgkin lymphomas [4].

Its genesis is linked to genetic mutations of proto-oncogenes and tumor suppressor genes, viral infections such as HIV, EBV, and human herpes virus-8 (HHV-8), and the presence of a lymph node environment that promotes lymphomagenesis. It can also arise from the transformation of low-grade B-cell lymphomas, such as splenic marginal zone lymphoma, and from the transformation of chronic lymphocytic leukemia (Richter transformation) [5,6]. According to the gene expression profile, two molecular subtypes of DLBCL have been distinguished: The germinal center B-cell-like (GCB) and the activated B-cell-like (ABC) while approximately 10% to 15% of lymphomas remain unclassifiable [4].

The World Health Organization (WHO) defines high-grade B-cell lymphomas as lymphomas that present rearrangements in the MYC, BCL-2, and/or BCL-6 genes (double expressor/double hit or triple expressor/triple hit). These represent approximately 4% to 8% of cases of large B-cell lymphomas and have variable morphology, including DLBCL, unclassifiable B-cell lymphoma (with features intermediate between DLBCL and Burkitt's lymphoma), and lymphomas with blastoid features. This entity usually has an aggressive clinical presentation and presents a high risk of central nervous system (CNS) involvement. Likewise, it usually has a poor prognosis and does not usually have good results with the R-CHOP (rituximab plus cyclophosphamide, doxorubicin, vincristine, and prednisone) regimen; therefore, more intense immunotherapy regimens such as DA-EPOCH-R should be considered. Triple expressor lymphoma is a DLBCL that presents overexpression of the MYC, BCL-2, and BCL-6 genes, but that does not present chromosomal rearrangements and its incidence is unknown because this entity is a subcategory [4,6].

Extranodal lymphomas with spinal involvement occur in approximately 6.5% of patients with non-Hodgkin's lymphoma and are usually a late manifestation of disseminated disease. These tend to have a primary origin in epidural tissue in 0.1% to 3.3% of cases. Likewise, these can originate in paraspinal, paravertebral, and retroperitoneal tissues that enter the epidural space through the intervertebral foramina [7-9]. The most frequently compromised region is the thoracic spine, followed by the lumbar and cervical spine. This is due to the great length of the thoracic spine compared to the other segments and its ability to produce bulky disease in the thorax and abdomen due to its rich venous plexus [10]. However, its presence is not well-defined due to its rare occurrence.

Certain molecular types of lymphoma have a predisposition for the central nervous system such as lymphomas with rearrangements in the MYC, BCL-2, and/or BCL-6 genes, double expressor/hit, and triple expressor/hit [4,11]. The clinical presentation of these tumors is usually with lumbar pain and signs and symptoms of spinal cord compression such as a motor deficit, paresthesia, bladder and intestinal dysfunction, and cauda equina syndrome due to loss of spinal cord integrity [7]. There are reported cases in the medical literature of DLBCL with CNS involvement, among which double expressor lymphomas and triple hit lymphomas stand out [7-13]. However, to date, no cases of triple expressor lymphoma with spinal cord involvement have been reported. To our knowledge, this is the first case report of a triple expressor lymphoma with acute presentation of paraparesis due to compressive myelopathy.

At the moment, there are no standardized guidelines for the management of this entity. However, various management regimens have been proposed, including the DA-EPOCH-R regimen [14]. Several studies have demonstrated the superiority of DA-EPOCH-R compared to other regimens, such as R-CHOP, in patients with double-expressor/hit and triple-expressor/hit lymphoma [1,14,15]. On the other hand, decompressive surgery is indicated in cases of spinal cord compression, emerging neurological deterioration, refractoriness to medical management, and in order to perform a biopsy of the specimen [7,10]. The patient of the case presented a good response to the management with almost complete resolution of spinal symptoms.

The patient in this case had acute low back pain that worsened over time. After that, the patient presented weakness in the lower extremities, so an MRI was requested, which showed a mass around the T9 vertebra with contrast enhancement that was causing acute spinal cord compression. Therefore, the patient underwent emergency surgery followed by relief of neurological symptoms. Furthermore, the patient exhibited almost complete resolution of the lesions.

## Conclusions

Triple expressor lymphoma is a subtype of DLBCL that is highly aggressive and often involves adjacent structures. Due to its unusual presentation, and its predisposition for the CNS, a high suspicion of this entity is required for undetermined CNS lesions. Currently, there are no standard management guidelines for this entity, however, several studies have shown the superiority of the DA-EPOCH-R regimen over R-CHOP.
